# The effects of geographical accessibility to health facilities on antenatal care and delivery services utilization in Benin: a cross-sectional study

**DOI:** 10.1186/s12978-021-01249-x

**Published:** 2021-10-14

**Authors:** Mariam Tanou, Takaaki Kishida, Yusuke Kamiya

**Affiliations:** 1 Ministry of Infrastructure, Building Lamizana, 03BP7011 Ouagadougou, Burkina Faso; 2 Graduate School of International Cooperation Studies, Kobe University, 2-1 Rokkodai, Nada-ku, Kobe, 657-8501 Japan; 3 Faculty of Economics, Ryukoku University, 67 Tsukamoto-cho, Fukakusa, Fushimi-ku, Kyoto, 612-8577 Japan

**Keywords:** Geographical accessibility, Distance, Travel time, Altitude, Antenatal care, Delivery services, Geographic Information System, Benin

## Abstract

**Background:**

The world is making progress toward achieving maternal and child health (MCH) related components of the Sustainable Development Goals. Nevertheless, the progress of many countries in Sub-Saharan Africa is lagging. Geographical accessibility from residence to health facilities is considered a major obstacle hampering the use of appropriate MCH services. Benin, a country where the southern and northern parts belong to different geographical zones, has among the highest maternal mortality rate in the world. Adequate use of MCH care is important to save lives of women and their babies. This study assessed the effect of geographical accessibility to health facilities on antenatal care and delivery services utilization in Benin, with an emphasis on geographical zones.

**Methods:**

We pooled two rounds of Benin Demographic and Health Surveys (BDHS). The sample included 18,105 women aged 15–49 years (9111 from BDHS-2011/2012 and 8994 from BDHS-2017/2018) who had live births within five years preceding the surveys. We measured the distance and travel time from residential areas to the closest health center by merging the BDHS datasets with Benin’s geographic information system data. Multivariate logistic regression analysis was performed to estimate the effect of geographical access on pregnancy and delivery services utilization. We conducted a propensity score-matching analysis to check for robustness.

**Results:**

Regression results showed that the distance to the closest health center had adverse effects on the likelihood of a woman receiving appropriate maternal healthcare. The estimates showed that one km increase in straight-line distance to the closest health center reduces the odds of the woman receiving at least one antenatal care by 0.042, delivering in facility by 0.092, and delivering her baby with assistance of skilled birth attendants by 0.118. We also confirmed the negative effects of travel time and altitude of women’s residence on healthcare utilization. Nonetheless, these effects were mainly seen in the northern part of Benin.

**Conclusions:**

Geographical accessibility to health facilities is critically important for the utilization of antenatal care and delivery services, particularly in the northern part of Benin. Improving geographical accessibility, especially in rural areas, is significant for further use of maternal health care in Benin.

## Plain language summary

Maternal and neonatal mortality rates are still high in many countries in Sub-Saharan Africa. Antenatal care (ANC) visits and institutional delivery with skilled birth attendants are important to prevent maternal and neonatal deaths. Nevertheless, women’s utilization of ANC and delivery services has decreased recently in Benin, a country where the southern and northern parts belong to different geographical zones.

Geographical accessibility from residence to health facilities is considered a major obstacle hampering the use of appropriate maternal healthcare. This study assessed the effect of geographical accessibility on ANC and delivery services utilization in Benin by considering the geographical characteristics.

We used the two rounds of the Benin Demographic and Health Survey 2011/2012 and 2017/2018 and conducted regression analysis.

This study has three important findings: (1) We confirmed adverse effects of distance and travel time on the likelihood of a women receiving appropriate ANC and delivery services in Benin, but this effect was mainly observed in the northern part; (2) Distance and travel time to health facilities had a negative effect on the use of at least one ANC but no significant effect for four or more ANC; (3) Regarding the threshold of distance, we confirmed that women living within 5 km from the closest health center were more likely to use maternal healthcare compared to their counterparts.

In conclusion, geographical accessibility to health facilities is critically important for the utilization of antenatal care and delivery services, particularly in the northern part of Benin.

## Background

Maternal and child health (MCH) care is an important investment a country can make to build human capital and boost economic growth [1]. It should be emphasized that the world is making progress toward MCH improvement. The global maternal mortality ratio fell from 342 to 211 deaths per 100,000 live births between 2000 and 2017 [2]. Nevertheless, maternal, neonatal, and child mortality rates are still high in many countries in Sub-Saharan Africa. For instance, in the Benin Republic, a coastal country located in West Africa, the maternal mortality ratio was 391 per 100,000 live births in 2018 [3].

Maternal deaths particularly occur during labor, delivery, and the immediate postpartum period, with obstetric hemorrhage being the leading cause [4, 5]. Empirical studies have demonstrated that antenatal care (ANC) visits, institutional delivery with skilled birth attendants (SBA), and postnatal care are important to prevent maternal and newborn deaths [5, 6]. In Benin, women’s utilization of maternal health care services has decreased recently. The percentage of women receiving at least one ANC shifted from 86% in 2011 to 83% in 2018, and those visiting at least four ANCs, as recommended by the World Health Organization (WHO), declined from 58 to 52% during the same period [3]. Moreover, there are still a certain number of women who give birth at alternative places such as their homes and those of traditional birth attendants who are not knowledgeable in modern obstetric care [7].

Among the determinants of not utilizing appropriate maternal healthcare services in Sub-Saharan Africa, geographical accessibility to health facilities is considered a major obstacle [8–12]. Although several empirical studies on the issue of maternal healthcare service utilization in Benin have been conducted [7, 13–16], they mainly focused on the individual, household, and community levels. No study has analyzed the effects of geographical accessibility to health facilities on maternal healthcare utilization by using geographical information system (GIS) of Benin. Therefore, we conducted our study to analyze the effects of geographical accessibility to health facilities on antenatal care and delivery services utilization in Benin.

## Methods

### Study setting

Benin is located in West Africa on the Gulf of Guinea, and covers an area of 114.763 square km. Benin consists of 12 departments: Alibori, Atacora, Atlantique, Borgou, Collines, Couffo, Donga, Littoral, Mono, Ouémé, Plateau, and Zou [17]. These departments are divided into 77 municipalities and then subdivided into 546 districts [17]. In terms of maternal and neonatal healthcare, Benin has two national hospitals, six Departmental Hospital Centers, 28 Zone Hospitals, 12 other hospitals, 76 municipality maternity units and more than 825 district maternity units [18].

Benin is a country where the southern and northern parts belong to different geographical zones. Benin’s geographic gradient is well-marked from south to north. The southern part of the country is in the coastal zone, whereas the northern part is mountainous. The Atakora mountain chain culminates at 658 m above sea level. Regarding geographical accessibility within Benin, the main mode of transport used are two-wheelers (personal motorbikes and motorbike taxis) and personal cars for road transport [19]. The ownership rate of cars per household was low, at 4.0% in 2011/2012 and 3.9% in 2017/2018 [3]. On the other hand, the ownership rate of motorbikes per household was comparatively higher, increasing from 58.4% in 2011/2012 to 66.3% in 2017/2018 [3, 20]. Road infrastructure consists of “classified roads” (6076 km) and “rural roads” (23,000 km) [19]. “Classified roads” include national roads (3898 km) and interstate national roads (2178 km), but only 30% of “classified roads” are paved [19]. Regarding “rural roads”, only 1/3 (a total of 7400 km) are properly constructed [19]. The low percentage of asphalted roads in the country leads to the high ownership of intermediate means of transport among households, such as motorcycles, tricycles, animal-drawn carts, rickshaws, bicycles etc., which are flexible and efficient on rough roads [19, 21].

### Data

We used two latest cross-sectional data from the Benin Demographic and Health Survey (BDHS), 2017/2018 [3] and 2011/2012 [20]. Table 1 shows the summary of the surveys. Regarding the sampling design, in the first stage of BDHS 2017/2018, 555 primary sampling units (clusters) were drawn from the list of 12,633 enumeration areas [3]. In BDHS 2011/2012, 750 clusters were selected from 7352 enumeration areas [20]. In the second stage, 26 households and 24 households per cluster were selected in BDHS 2017/2018 and BDHS 2011/2012, respectively. BDHS 2017/2018 surveyed 14,156 households and 15,928 women [3], while BDHS 2011/2012 surveyed 17,422 households and 16,599 women [20]. We used data of 18,105 women, 8994 women from BDHS 2017/2018 and 9111 women from BDHS 2011/2012, who had live births within five years preceding the surveys as a study sample.

**Table 1:** Summary of the surveys

	BDHS 2017/2018	BDHS 2011/2012
Primary Sampling Units (clusters)	555	750
Enumeration areas	12,633	7352
Households per cluster	26	24
Households surveyed	14,156	17,422
Women surveyed	15,928	16,599

Our main explanatory variable is geographical accessibility. Geographical accessibility is defined as “the physical distance or travel time from the service delivery point to the user” [22]. In addition, several studies have treated the altitude of women’s residence as a significant aspect of geographical accessibility [23, 24]. Accordingly, our study analyzed distance, travel time, and the altitude of women’s residence as important aspects of geographical accessibility. We used the GIS module of the BDHS and the ArcGIS software to calculate these variables.

First, we used the “Euclidean distance (km)”—a straight-line distance between two points—to measure the distance from one’s residence to the closest health facility [25]. The advantage of using this method is that it can be generalized for other similar topography and cultural contexts in Sub-Saharan Africa [25]. Several previous studies used the Euclidean distance to estimate geographical accessibility to health facilities in Sub-Saharan Africa [12, 26, 27]. However, using straight-line distance to assess geographical accessibility is sometimes regarded as less accurate than applying travel distance because it is the simplest measure [28–30]. In addition, Euclidean distance may lead to an underestimation of travel distance [26]. Despites these limitations, Euclidean distance is considered a valid measure of accessibility in both rural and urban areas in Sub-Saharan Africa [25, 31].

Second, we used the “road network distance” as a more realistic measure. It is the distance along road infrastructure from a residence to the closest health facility. It can be defined as the Euclidean distance from the residence to the road network, plus a distance from the road network to a health facility [25]. To estimate the road network distance, we used the Benin Road Infrastructure dataset from the World Bank website that contains shapefiles of all roads as of 2017 [32]. We also used the geolocation points of the clusters provided by the BDHS and those of health facilities from the World Health Organization (WHO) website [33].

Third, we calculated travel time (in minutes: by walking and via car) from a residence to the closest health facility. The walking time was calculated from the women’s residence to the closest health center, following paths and roads for pedestrians. Walking speed was set at 5 km/h. Considering the types of roads, the driving time was calculated from the women’s residence to the closest health center. Driving speed was set at 80 km/h for “classified roads” and 60 km/h for “rural roads.” We used Dijkstra’s algorithm of ArcGIS, which can estimate the driving time from the residence to the closest health center by using the fastest route.

Fourth, we examined the effects of the geographic altitude of a residence (in meters) on women’s utilization of maternal healthcare resources. It was assumed that it would be more difficult for women living in highland areas to reach health facilities as confirmed in Ethiopia [23]. We used ArcGIS to conduct the abovementioned analysis.

Finally, we analyzed whether the availability of means of transport at the community level was associated with maternal healthcare utilization. The BDHS contains questions about whether the household owned a bicycle or motorbike. Because bicycles and motorbikes are popular means of transport in Benin, they can be used to travel when seeking maternal healthcare at health facilities [34]. Since the ownership of motorbikes and bicycles at the household level seems less important than its availability, we used the ownership rates of them per cluster (community), which would reflect more realistic situation of Benin. Thus, we calculated the ownership rates of bicycles and motorbikes per cluster and used them as proxy variables for community-level availability of means of transport.

### Statistical analysis

We applied multivariate logistic regressions to analyze the impact of distance to the closest health center on maternal healthcare utilization. Data analysis was performed using Stata version 14. Because BDHS applied a two-stage cluster sampling design, we used the svy (survey) commands of Stata to correct for unequal sampling probability, clustering, and stratification to calculate descriptive statistics and perform regression analysis. Additionally, we conducted propensity score matching (PSM) analysis to check the robustness of the logistic regressions. The PSM attempts to estimate the effects of a specific policy or treatment in observational studies by reducing the bias arising from confounding factors that might predict outcome variables. It matches treated and untreated units based on a set of basic characteristics and attempts to balance both groups. According to the National Health Development Plan (NHDP) 2009–2018 of Benin, geographical accessibility to healthcare services in Benin was defined as “the percentage of the population living within 5 km of the closest health center” [35]. Therefore, we created two groups, the treatment group (women living more than 5 km from the closest health center) and a comparison group (women living within 5 km from the closest health facility) to assess whether the threshold (5 km from the closest health center) had adverse effects on women’s use of appropriate maternal healthcare.

### Outcome variables

We used the following outcome variables: (1) whether the woman made at least one ANC visit during her latest pregnancy (“any ANC”); (2) whether the woman made four or more ANC visits during her latest pregnancy (“ ≥ 4 ANC”); (3) whether the woman used a health facility at the birth-delivery (“Facility delivery”); and (4) whether the woman was attended by a professional health worker (i.e., doctor, nurse, auxiliary nurse, or midwife at birth (“delivery by SBA”). Because all the outcome variables were binary, they were coded 1 if the mother had received appropriate healthcare (ANC, facility delivery, or SBA) during pregnancy or childbirth, and 0 otherwise.

### Control variables

We used mother-, household-, and community-level characteristics as control variables. Mother-level variables comprised age and educational achievement (no education, primary, secondary/higher). Household-level variables included religion of the household head (Muslim, Protestant, Catholic, Vodoum/other traditional, and No religion/others) and asset quintiles. Community-level variables included geographical zones of a residence (south or north of the country), as well as the ownership rates of bicycles and motorbikes.

## Results

### Sample characteristics

Table 2 summarizes the characteristics of the study participants. After merging two rounds of BDHS, 18,105 women (49.9% from BDHS 2011/2012 and 50.1% from BDHS 2017/2018) and 26,996 births were included in this study. Regarding geographical zones, 55.7% of the sample (10,087 women) represented southern, while 44.3% (8018 women) represented the northern part of the country. Regarding outcome variables, 84.0% of the women received ANC at least once, and 54.8% received ANC four times or more. Of all the childbirth, 86.0% were delivered at health facilities, and 81.7% were assisted by SBA. These figures differed significantly between the geographical zones. Although 92.0% and 68.4% of the women received at least one ANC and four or more ANC in the South, only 73.3% and 36.8% of the women received the same care in the North. Regarding childbirth, 95.1% of the babies were born at health facilities and 92.0% were assisted by SBA in the South, but only 73.8% and 68.1% of the newborns received the same care in the North.

**Table 2:** Sample characteristics

	Total			South			North		
Variables	Obs	Mean	S.D	Obs	Mean	S.D	Obs	Mean	S.D
Outcome variables									
Received any ANC	17,489	0.840	0.367	9659	0.920	0.271	7830	0.733	0.442
Received ≥ 4 ANC	17,489	0.548	0.498	9659	0.684	0.465	7830	0.368	0.482
Facility delivery	26,950	0.860	0.347	14,989	0.951	0.216	11,961	0.738	0.440
Delivery by SBA	26,996	0.817	0.386	15,012	0.920	0.271	11,984	0.681	0.466
Main predictor									
Straight-line distance (km)	17,768	3.57	4.37	9750	2.26	2.08	8018	5.29	5.78
Road network distance (km)	17,325	4.85	6.21	9394	3.14	3.29	7931	7.10	8.14
Travel time by walk (minutes)	17,325	58.2	74.5	9394	37.7	39.5	7931	85.2	97.6
Travel time by car (minutes)	17,325	8.1	11.8	9394	5.3	6.1	7931	11.8	15.8
Altitude (meter)	18,018	171.4	148.2	10,087	63.0	64.6	7931	314.2	97.6
Distance dummy: 5 km or more	17,768	0.197	0.397	9750	0.068	0.251	8018	0.367	0.482
Mothers' characteristics									
Age	18,105	29.3	6.8	10,087	29.6	6.6	8018	28.9	7.0
Education									
No education	18,105	0.673	0.469	10,087	0.600	0.490	8018	0.777	0.416
Primary	18,105	0.175	0.380	10,087	0.214	0.410	8018	0.121	0.326
Secondary/Higher	18,105	0.151	0.358	10,087	0.186	0.389	8018	0.103	0.303
Households' characteristics									
Religion									
Muslim	18,105	0.286	0.452	10,087	0.085	0.279	8018	0.569	0.495
Protestant	18,105	0.278	0.448	10,087	0.398	0.490	8018	0.109	0.312
Catholic	18,105	0.252	0.434	10,087	0.300	0.458	8018	0.184	0.387
Vodoum/other traditional	18,105	0.114	0.318	10,087	0.158	0.365	8018	0.053	0.223
No religion/others	18,105	0.070	0.255	10,087	0.058	0.234	8018	0.085	0.280
Asset									
Lowest	18,105	0.202	0.401	10,087	0.128	0.334	8018	0.306	0.461
Lower middle	18,105	0.201	0.401	10,087	0.167	0.373	8018	0.250	0.433
Middle	18,105	0.201	0.401	10,087	0.189	0.392	8018	0.217	0.412
Upper middle	18,105	0.203	0.402	10,087	0.238	0.426	8018	0.154	0.361
Highest	18,105	0.193	0.394	10,087	0.278	0.448	8018	0.073	0.260
Community's characteristics									
Ownership rate									
Bicycle	17,878	0.300	0.207	9977	0.227	0.175	7901	0.404	0.205
Motorbike	17,878	0.624	0.177	9977	0.600	0.182	7901	0.658	0.163
Round									
BDHS 2011/2012	18,105	0.499	0.500	10,087	0.560	0.496	8018	0.414	0.493
BDHS 2017/2018	18,105	0.501	0.500	10,087	0.441	0.496	8018	0.586	0.493

Regarding the main predictor, the mean straight-line distance from a residence to the closest health facility was 3.57 km in the total sample. The distance in the north (5.29 km) was twice as long as that in the South (2.26 km). Looking at as a distance dummy variable, 19.7% of the households lived more than 5 km away from the closest health center in the total sample. The percentage was almost five times higher in the North (36.7%) than in the South (6.8%).

Regarding mothers’ characteristics, their mean age was 29.3 years. Of all the mothers, 67.3% were not formally educated; only 15.1% had completed secondary education or higher. As for household characteristics, 28.6% of the household heads were Muslim, followed by Protestants (27.8%), Catholics (25.2%), Vodoum/other traditional religion (11.4%), and no religion/others (7.0%). In terms of community characteristics, the ownership rates of bicycles and motorbikes per cluster were 30.0% and 62.4%, respectively.

### Regression analysis

Table 3 present the results of the multivariate logistic regression analysis for antenatal care and delivery services utilization. The magnitude of the effects was assessed by odds ratio (OR), which can be interpreted as increasing (if OR > 1) or reducing (if OR < 1) the likelihood of women’s use of healthcare. Regarding the main predictor (straight-line distance to the closest health center in the Model 1), there was a statistically significant and negative effect of having any ANC, facility delivery, and SBA, indicating that the longer the distance to the closest health center, the less likely women are to receive the necessary healthcare during pregnancy and childbirth. The estimated ORs for any ANC (OR = 0.958, *p* < 0.001) indicated that if the straight-line distance to the closest health center increased by one km, the odds of a woman receiving ANC at least once was reduced by 0.042. Similarly, the estimated ORs for facility delivery (OR = 0.908, *p* < 0.001) and SBA (OR = 0.882, *p* < 0.001) suggest that one-kilometer increase in straight-line distance decreases the odds of delivering a baby at a health facility by 0.092 and reduces the odds of being assisted by SBA by 0.108. However, these results differed between geographical zones. In the South, a statistically significant and negative effect of distance on the use of maternal healthcare was not confirmed. On the other hand, distance had statistically negative effects on women’s uptake of ANC (OR = 0.955, *p* < 0.001), facility delivery (OR = 0.894, *p* < 0.05), and SBA (OR = 0.879, *p* < 0.001) in the North. Table 3 shows statistically significant and negative effects of travel distance (Model 2), travel time by walk (Model 3) and travel time by car (Model 4) on women’s uptake of any ANC, facility delivery, and SBA.

**Table 3:** Results of multivariate logistics regressions for antenatal care and delivery services utilization

	Total				South				North			
	Any ANC	≥ 4 ANC	Facility	SBA	Any ANC	≥ 4 ANC	Facility	SBA	Any ANC	≥ 4 ANC	Facility	SBA
Model 1												
Straight-line distance (km)	0.958 (0.000)***	0.998 (0.747)	0.908 (0.000)***	0.882 (0.000)***	0.974 (0.443)	1.013 (0.448)	0.955 (0.553)	0.859 (0.051)	0.955 (0.000)***	0.995 (0.525)	0.894 (0.000)***	0.879 (0.000)***
Altitude	0.999 (0.045)*	0.998 (0.000)***	0.991 (0.000)***	0.994 (0.000)***	0.999 (0.403)	0.999 (0.023)*	0.989 (0.000)***	0.990 (0.000)***	0.999 (0.055)	0.998 (0.000)***	0.992 (0.000)***	0.996 (0.012)*
Mother-level												
Age	1.004 (0.202)	1.007 (0.014)*	0.965 (0.000)***	0.971 (0.001)***	1.000 (0.940)	1.002 (0.561)	0.974 (0.115)	0.979 (0.189)	1.007 (0.113)	1.012 (0.003)**	0.963 (0.001)***	0.970 (0.003)**
Education												
No education†												
Primary	1.935 (0.000)***	1.433 (0.000)***	2.985 (0.000)***	2.012 (0.000)***	1.697 (0.000)***	1.375 (0.000)***	2.890 (0.001)**	1.613 (0.080)	2.109 (0.000)***	1.535 (0.000)***	3.040 (0.000)***	2.428 (0.001)**
Secondary/Higher	1.670 (0.000)***	1.828 (0.000)***	9.254 (0.000)***	2.951 (0.000)***	1.158 (0.350)	1.683 (0.000)***	5.727 (0.002)**	1.652 (0.161)	2.361 (0.000)***	2.053 (0.000)***	12.444 (0.000)***	5.235 (0.000)***
Household-level												
Religion												
Muslim†												
Protestant	2.590 (0.000)***	1.509 (0.000)***	3.404 (0.000)***	3.340 (0.000)***	1.715 (0.009)**	1.698 (0.000)***	1.263 (0.676)	1.301 (0.569)	2.985 (0.000)***	1.400 (0.001)***	5.213 (0.000)***	6.932 (0.000)***
Catholic	2.587 (0.000)***	1.389 (0.000)***	4.452 (0.000)***	3.621 (0.000)***	1.835 (0.007)**	1.626 (0.000)***	1.708 (0.369)	1.254 (0.632)	2.731 (0.000)***	1.277 (0.005)**	5.335 (0.000)***	5.005 (0.000)***
Vodoum/other traditional	1.512 (0.002)**	1.087 (0.341)	0.926 (0.791)	0.698 (0.205)	1.052 (0.839)	1.207 (0.141)	0.372 (0.112)	0.440 (0.106)	1.488 (0.047)*	1.094 (0.584)	1.118 (0.783)	0.356 (0.031)*
No religion/others	1.260 (0.065)	1.001 (0.989)	1.392 (0.218)	1.313 (0.306)	0.838 (0.441)	1.225 (0.185)	0.819 (0.789)	0.750 (0.598)	1.369 (0.038)*	0.919 (0.504)	1.477 (0.171)	1.271 (0.448)
Asset												
Lowest†												
Lower middle	1.850 (0.000)***	1.482 (0.000)***	2.214	2.412 (0.000)***	1.588 (0.000)***	1.310 (0.001)***	1.845 (0.029)*	2.198 (0.004)**	1.941 (0.000)***	1.600 (0.000)***	2.338 (0.000)***	2.371 (0.000)***
			(0.000)***									
Middle	2.530 (0.000)***	2.010 (0.000)***	4.471 (0.000)***	3.580 (0.000)***	2.224 (0.000)***	1.827 (0.000)***	3.410 (0.000)***	4.074 (0.000)***	2.607 (0.000)***	2.097 (0.000)***	4.989 (0.000)***	3.063 (0.000)***
Upper middle	5.040 (0.000)***	2.858 (0.000)***	16.482 (0.000)***	13.311 (0.000)***	4.015 (0.000)***	2.512 (0.000)***	18.157 (0.000)***	25.160 (0.000)***	5.552 (0.000)***	3.167 (0.000)***	14.329 (0.000)***	7.255 (0.000)***
Highest	5.408 (0.000)***	4.348 (0.000)***	68.833 (0.000)***	23.592 (0.000)***	3.907 (0.000)***	4.002 (0.000)***	34.298 (0.000)***	33.036 (0.000)***	9.931 (0.000)***	4.945 (0.000)***	203.151 (0.000)***	18.295 (0.000)***
Community-level												
Ownership rate												
Bicycle	0.863 (0.546)	0.930 (0.608)	0.331 (0.072)	0.054 (0.000)***	0.670 (0.326)	0.863 (0.480)	0.437 (0.420)	0.086 (0.016)*	0.967 (0.911)	1.008 (0.966)	0.353 (0.189)	0.058 (0.001)**
Motorbike	1.908 (0.014)*	0.875 (0.370)	11.272 (0.001)***	13.112 (0.000)***	1.491 (0.316)	0.666 (0.029)*	3.133 (0.256)	2.439 (0.336)	2.332 (0.018)*	1.410 (0.162)	52.379 (0.000)***	121.161 (0.000)***
Round												
BDHS 2011/2012†												
BDHS 2017/2018	1.011 (0.903)	0.682 (0.000)***	0.807 (0.366)	0.377 (0.000)***	1.151 (0.343)	0.776 (0.000)***	0.608 (0.164)	0.209 (0.000)***	0.955 (0.688)	0.576 (0.000)***	1.117 (0.723)	0.748 (0.389)
Observation	16,945	16,945	26,147	26,193	9232	9232	14,343	14,343	7713	7713	11,804	11,804

Model 2												
Travel distance (km)	0.973 (0.000)***	0.996 (0.392)	0.939 (0.000)***	0.927 (0.000)***	0.980 (0.290)	0.991 (0.335)	0.948 (0.217)	0.947 (0.237)	0.972 (0.000)***	0.998 (0.700)	0.933 (0.000)***	0.920 (0.000)***
Altitude	0.999 (0.042)*	0.998 (0.000)***	0.991 (0.000)***	0.994 (0.000)***	0.999 (0.307)	0.999 (0.035)*	0.989 (0.000)***	0.989 (0.000)***	0.999 (0.054)	0.998 (0.000)***	0.992 (0.000)***	0.996 (0.017)*
Observation	16,528	16,528	25,494	25,539	8900	8900	13,823	13,845	7628	7628	11,671	11,694
Model 3												
Travel time by walk (minutes)	0.998	1.000	0.995	0.994	0.998	0.999	0.996	0.995	0.998	1.000	0.994	0.993
	(0.000)***	(0.392)	(0.000)***	(0.000)***	(0.290)	(0.335)	(0.217)	(0.237)	(0.000)***	(0.700)	(0.000)***	(0.000)***
Altitude	0.999	0.998	0.991	0.994	0.999	0.999	0.989	0.989	0.999	0.998	0.992	0.996
	(0.042)*	(0.000)***	(0.000)***	(0.000)***	(0.307)	(0.035)*	(0.000)***	(0.000)***	(0.054)	(0.000)***	(0.000)***	(0.017)*
Observation	16,528	16,528	25,494	25,539	8900	8900	13,823	13,845	7628	7628	11,671	11,694
Model 4												
Travel time by car (minutes)	0.985 (0.000)***	0.997 (0.239)	0.965 (0.000)***	0.960 (0.000)***	0.986 (0.111)	0.994 (0.180)	0.960 (0.094)	0.976 (0.342)	0.985 (0.000)***	0.999 (0.621)	0.965 (0.001)***	0.955 (0.000)***
Altitude	0.999 (0.033)*	0.998 (0.000)***	0.991 (0.000)***	0.994 (0.000)***	0.999 (0.288)	0.999 (0.031)*	0.989 (0.000)***	0.989 (0.000)***	0.999 (0.048)*	0.998 (0.000)***	0.992 (0.000)***	0.996 (0.016)*
Observation	16,528	16,528	25,494	25,539	8900	8900	13,823	13,845	7628	7628	11,671	11,694

Odds ratios (ORs) are reported, *p < 0.05 **p < 0.01 ***p < 0.001, †: Reference, Zone dummies were also included as control variables, Estimates of the covariates for Model 2-4 are omitted from the table

With respect to the other explanatory mother and household level variables, mothers’ educational attainment and household assets were consistently associated with a higher likelihood of utilizing ANC, facility-based delivery, and SBA. Regarding religion, women whose heads of household are Protestant and Catholic were more likely to receive appropriate MCH care services compared to Muslims. As for the community-level variables, the ownership rate of motorbikes was associated with a higher likelihood of having facility-based delivery and SBA in the North.

Table 4 presents the PSM results. In the total sample, the treatment dummy variable had a statistically significant and negative effect on receiving any ANC (OR = 0.952, *p* < 0.001), facility delivery (OR = 0.69, *p* < 0.001) and delivery by SBA (OR = 0.973, *p* < 0.001), indicating that women who lived 5 km or more from the closest health center were less likely to use these services compared to the comparison group. The negative effects of distance on receiving any ANC (OR = 0.943, *p* < 0.001), and delivery by SBA (OR = 0.973, *p* = 0.008) were confirmed in the southern part of the country. In north, the distance had statistically and negative effects on women’s uptake of ANC (OR = 0.965, *p* = 0.003), facility delivery (OR = 0.957, *p* < 0.001), and SBA (OR = 0.969, *p* = 0.003).

**Table 4:** Results of propensity score matching estimations

	Received any ANC	Received ≥ 4 ANC	Facility delivery	Delivery by SBA
Total				
Treatment dummy: 5 km or more from the health center	0.952 (0.000)***	0.982 (0.201)	0.969 (0.000)***	0.973 (0.000)***
Observation	16,495	16,495	26,147	26,193
South				
Treatment dummy: 5 km or more from the health center	0.943 (0.000)***	0.993 (0.715)	0.973 (0.008)**	0.988 (0.214)
Observation	9232	9232	14,343	14,366
North				
Treatment dummy: 5 km or more from the health center	0.965 (0.003)**	0.998 (0.887)	0.957 (0.000)***	0.969 (0.003)**
Observation	7713	7713	11,804	11,827

Odds ratios (ORs) are reported, *p < 0.05 **p < 0.01 ***p < 0.001, p-values in brackets

## Discussion

We analyzed the effects of geographical accessibility to health facilities on the use of antenatal care and delivery services in Benin utilizing a national-representative sample from two rounds of BDHS datasets along with GIS data on health center locations. We confirmed that geographical accessibility to the closest health center, after controlling for potential confounders, had negative effects on the use of maternal healthcare services except for at least four ANCs. This adverse effect of geographical access on maternal healthcare utilization is consistent with the results of numerous previous studies in Sub-Saharan African countries [11, 12, 27, 36, 37].

There are four points to be discussed here. First, although we confirmed the adverse effect of geographical access on maternal healthcare utilization in Benin, this effect was mainly observed in the northern part. It is recognized that the North of Benin is more rural and less equipped with health infrastructure than the South [13]. For instance, three departments in the southern part—Atlantique, Ouémé, and Littoral— encompass 48 percent of all 1155 private health facilities in the country [38]. Thus, expecting women in the South generally have easier access to maternal healthcare compared to their counterparts in the North. On the other hand, the average distance to the closest health center was longer, and the percentage of women using appropriate maternal healthcare was actually lower in the North (Table 2). Besides, In Northern Benin, road infrastructure is less developed, and the population is more dispersed than in the South [13, 39]. Thus, geographical access to health facilities becomes critically important to receive the necessary maternal healthcare in the North. A previous study conducted in Benin found consistent difficulties in accessing health facilities for a birth delivery among women in the North [13]. We also confirmed that travel time to the closest health centers had negative effects on women’s use of healthcare services in line with the numerous studies conducted in Sub-Saharan Africa countries [37, 40].

In general, the negative impact of distance on healthcare utilization increases when it is combined with a lack of transportation in developing countries [41, 42]. Regarding means of transport, Table 2 shows that the community’s ownership rates of bicycles and motorbikes were higher in the North than in the South, implying that these vehicles are more common transport means for people in the northern part. The results of the logistic regressions showed that the ownership rate of motorbikes had positive effects on women’s uptake of facility delivery and SBA in the North (Table 3). Since a higher ownership rate of motorbikes per cluster also indicates better road conditions of the community [19], transport infrastructure is critical for women’s use of maternal healthcare. Previous studies in Mali and Nepal showed similar results in that poor road conditions reduced the likelihood of receiving timely ANC [24, 43].

Second, our finding that distance to health facilities had a negative effect on the use of at least one ANC but no effect for four or more ANC is consistent with previous studies in Tanzania [29], Zambia [26], and Ethiopia [44]. However, other empirical studies conducted in many African countries found the adverse effects of distance on women’s uptake of four or more ANC visits [37]. Regarding this point, empirical studies in Nigeria [45] and other low- and middle-income countries [46] reported some other factors influencing women’s uptake of four or more ANC regardless of distance such as absence of good medication and health workers, disparity between the nature of antenatal provision and the expectations of the women. In Benin, the persistent shortage of healthcare workers leads to low performance of health facilities and quality of healthcare, leading to lowered motivation to go to healthcare facilities [13, 47].

Third, regarding the threshold of distance, our PSM analysis confirmed that women living within 5 km from the closest health center were more likely to use maternal healthcare compared to their counterparts. This result is consistent with the systematic review of 31 empirical studies that showed that living within 5 km of obstetrical facilities was significantly associated with a higher likelihood of delivering a baby at a health facility [42]. A study in Haiti also showed that the availability of health centers within 5 km of a residence increased the odds of receiving ANC services [48]. Our study confirmed the appropriateness of the definition of “geographical accessibility to healthcare” by Benin’s NHDP 2009–2018 as the percentage of the population living within 5 km from the closest health center [17].

Fourth, we found that the geographical altitude of a residence had a negative effect on the utilization of ANC and delivery services. Previous studies conducted in low-income countries showed that altitude lead to inequalities in the use of MCH services. In Ethiopia, a difference in altitude between home and health facilities was confirmed to be associated with a smaller proportion of women using maternal health care services [23]. In Nepal, a study showed that women residing in mountainous areas had difficulty accessing institutional delivery [24]. Therefore, it indicates that even though the health facility is located closer to one’s residence, it needs more effort to be reached especially for women in labor.

As policy implication of our study, firstly, it is critical to improve the geographical accessibility from the women’s residences to health facilities for further use of maternal healthcare in Benin. In particular, the government of Benin needs to create strategies that facilitate transportation for women living in remote areas to access health facilities. For instance, introducing alternative transportation such as “motorcycle ambulances” used in Malawi [49] and Uganda [50] can potentially reduce the difficulties women face while traveling. In Malawi, motorcycle ambulances are stationed at remote rural health centers. The ambulances are operated by the trained health surveillance staff and transport women seeking maternal healthcare from their homes to health facilities. The transportation provided is free of charge. Motorcycle ambulances are cheaper and more effective alternatives in terms of purchasing and operating costs than car ambulances [49, 51]. Secondly, Benin’s current Health Development Plan considers distance from a residence to health facilities as a single indicator of “geographical accessibility to healthcare.” [35] Our study confirmed the negative effect of geographical altitude on maternal healthcare utilization; the altitude of women’s residence should also be treated as an important aspect of geographical accessibility.

Our research was not without limitations. In estimating the Euclidean distance and road network distance, we could not consider physical features, rivers to be crossed, rainforests, rocky sections, and plains. These geographic features should be analyzed as additional and important factors hindering maternal healthcare utilization in a future study.

## Conclusions

We confirmed that geographical access to the closest health center, measured as distance (both as straight-line distance and road network distance), travel time, and altitude of women’s residence, had a significant and negative effect on women’s use of antenatal care and delivery services, mainly in the northern part of Benin. Improving geographical accessibility, especially in rural areas, is significant for further use of maternal healthcare in Benin.

## Data Availability

The dataset used during the current study are in the public domain and can be obtained from the DHS Program (http://dhsprogram.com//) or from the corresponding author on reasonable request.

## Abbreviations

ANC: Antenatal care
BDHS: Benin Demographic and Health Survey
GIS: Geographic Information System
MCH: Maternal and child health
NHDP: National Health Development Plan
OR: Odds ratio
PSM: Propensity score matching
SBA: Skilled birth attendant
WHO: World Health Organization
